# Tubcrculosis in Cattle

**Published:** 1895-11-23

**Authors:** 


### TUBERCULOSIS IN CATTLE.

Professor R. Wallace has written to the *Times* urging the adoption of what he calls "the only feasible proposal" which has been made in the direction of stamping out tuberculosis among our herds.

Shortly, it is as follows: (1) To compulsorily inoculate all suspected herds with tuberculin twice annually, separating those which give the reaction from the healthy, and destroying all unthrifty diseased animals; (2) to sell as food the flesh of such cattle as appeared healthy, notwithstanding that they might have responded to the tuberculin test, if, on examination, it were found to be such as would pass the sanitary market inspector at the present time.

All the compensation which the Government would be called upon to pay would be: (1) The value of the condemned cattle; and (2) the difference between the original value and the forced sale value of all that were slaughtered in the public interest, but whose flesh was sold.

Although we are very strongly impressed with the danger to man involved in the prevalence of tuberculosis in cattle, and, in fact, because we look upon the adoption of proper measures for its mitigation as a matter of the greatest importance, we think it well to point out how inoperative such a scheme would probably be in attaining the object for which it is put forward.

The first objection is that no measures which can be devised for the limitation of tuberculosis among our herds can take away or lessen the necessity of instituting a complete inspection of meat before it is allowed to be used as food, and that if such inspection is consistently and properly carried out the sale of tuberculous meat would become an impossibility, which, so far as the public is concerned, is the main thing to be aimed at. But the second and great objection to any system of slaughter and compensation is, that such a system, instead of stamping-out tuberculosis, might tend even to encourage its extension.

The farmer's aim is that every cow should ultimately go to the butcher, and that in the meantime she should give as much milk as possible; and so long as he has the dread of the slaughter-house inspector before his eye it is to his interest that every doubtful beast should be got rid of before the disease is so advanced as to make it likely that the animal will be condemned. But with a sure market by way of compensation his interest would be all the other way. Nor would there lie upon the farmer any urgent pressure to reform his cowsheds so long as Government would compensate him for every animal attacked by tuberculosis, notwithstanding that it might be entirely owing to his neglect and the condition into which he had allowed his sheds to drift that the malady had attacked the beasts at all. The evidence is so overwhelming that the condition of the cowsheds is at the root of a great deal of the tuberculosis which exists among our cattle, that it seems quite out of the question to relieve the farmers of responsibility in the matter. Hard as it may seem to refuse compensation to the farmer, we cannot shut our eyes to the fact that public interest is involved in the meat and milk rather than in the cows, and that it is the farmer's business, rather than that of the Government, to discover how meat and milk can be produced of such quality as to pass the standard. While, however, we think that the public should demand a rigid inspection of meat and a careful supervision of the whole trade of milk production, making it illegal to sell any that is not sound, we would also, both for the protection of the consumer and in common fairness to the farmer, insist upon the most careful investigation of all dead meat imported from other countries. The magnitude of this trade is very great, and in relation to it we may state that during last month while 17,931 tons of meat were received at the London Central Markets from the provinces, 4,057 came from abroad, 5,725 from America, and 4,623 from Australia and New Zealand. Thus nearly half the meat received came from other than English sources, and should in all fairness be subjected to an inspection quite as rigid as may be enforced on that produced in England.

